# Patient needs in the context of gynecologic oncology and breast cancer: a validation study

**DOI:** 10.1038/s41598-025-20816-x

**Published:** 2025-09-24

**Authors:** Susanne Theis, H. Frühwein, A. Hasenburg, M. Moehler, N. W. Paul

**Affiliations:** 1 Department of Obstetrics and Gynecology, University Medical Center of Johannes Gutenberg University Mainz, Langenbeckstraße 1, 55131 Mainz, Germany; 2 Institute of History, Philosophy, and Ethics of Medicine, Johannes Gutenberg University Medical Center Mainz, Am Pulverturm 13, 55131 Mainz, Mainz, Germany; 3 Department of Internal Medicine I, University Medical Center of Johannes Gutenberg University Mainz, Langenbeckstraße 1, 55131 Mainz, Germany

**Keywords:** Needs, Clinical ethics, Gynecologic oncology, Breast cancer, Patient centered care, Qualitative research, Grounded theory, Cancer, Health care, Oncology

## Abstract

Medical advancements and therapeutic innovations are progressing rapidly, necessitating corresponding adaptations in the delivery of patient-centered care. This study explores the needs of women diagnosed with gynecologic and breast cancers, using the frameworks of the National Academy of Medicine and the Picker Institute as reflective lenses. We conducted a qualitative, single-center study employing grounded theory methodology. Semi-structured interviews were carried out with 20 patients undergoing treatment for gynecologic malignancies and breast cancer at a university medical center. Interviews covered multiple phases of illness, including treatment initiation, adaptation, abstention, and future-oriented decisions. Data were analyzed inductively using constant comparative methods. Findings largely affirmed the relevance of the eight established domains of patient-centered care - personal values, clear information and education, emotional support, involvement of family and friends, physical comfort, coordinated care, continuity, and access to treatment. Three additional insights emerged: needs vary according to illness phase and tend to taper over time; they are grounded in ethical dimensions, including social identity, epistemic agency, and moral distress; and they are interdependent, requiring holistic rather than isolated responses. These results highlight that effective patient-centered care must account not only for the content of care but also for its timing, context and ethical significance, warranting integration into standard oncological practice.

**Supplementary Information:**

The online version contains supplementary material available at 10.1038/s41598-025-20816-x.

## Background

As medical possibilities and therapeutic options evolve rapidly, care for women with gynecologic cancers and breast cancers must keep pace with their lived realities. Patient-centered care (PCC) has become a hallmark of high-quality oncology because treatment choices intertwine with identity, fertility, family roles, body image, and long-term uncertainty^1^. Yet patients still report gaps between what matters to them and what systems deliver in everyday practice, including unmet supportive-care needs and uneven implementation of PCC in oncologic settings^2^.

Multiple frameworks have shaped PCC. The National Academy of Medicine (NAM; formerly IOM) established patient-centeredness as a core dimension of quality. System-level requirements such as respect for values and preferences, coordination and integration of care, information/communication/education, physical comfort, emotional support, and involvement of family and friends are articulated^3^. Complementing this, the Picker-Gerteis patient-experience work foregrounds what patients consistently value in care encounters^4,5^. Communication- and system-focused approaches e.g., Planetree, the Chronic Care Model, the Calgary-Cambridge guides, narrative-based medicine, and shared decision-making, help operationalize these principles in practice^6–15^. Despite this rich landscape, translation into routine oncology remains uneven, especially as precision oncology, workload pressures, and organizational constraints complicate trust, continuity, and individualized care^16–20^.

Two gaps are salient. First, ethically nuanced, qualitative evidence from European oncology remains limited compared with North American and Australasian contexts, including for women navigating reproductive cancers^21,22^. Second, the needs of women with gynecologic cancers and breast cancer are often treated as static, while clinical experience and emerging research suggest they vary by illness phase and carry moral dimensions—recognition and dignity, relational autonomy, epistemic balance (including the right not to know) and stigma^23–27^. Addressing these gaps requires patient-informed accounts that link the content of care to its timing, context, and ethical significance^16–18,22,24^.

Therefore, we conducted a qualitative study using interviews analyzed using grounded theory with women receiving treatment for gynecologic or breast cancer in a European academic center, examining how expressed needs map onto - and extend - the Picker-Gerteis patient-experience domains with attention to phase-specific variation. Our goal was to provide a preliminary validation of ethically salient needs in this setting and to indicate where existing PCC frameworks warrant temporal and ethical refinement^3–5,22,28^.

## Methods

### Study design

This qualitative sub-study is embedded within a broader research project funded by the German Cancer Aid Foundation (Deutsche Krebshilfe), which aims to reconstruct patients’ personal values, needs, therapeutic goals, and their expectations - both positive and negative - regarding social participation, identity, and self-perception during cancer treatment.

The study was prospectively registered with both the Research registry (Registration ID: 6615128527f813002936e32b) and the German Clinical Trials Register (DRKS00033242). Ethics approval was obtained from the Ethics Committee of the State Medical Association of Rhineland-Palatinate, Germany (approval no. 2022–16670, version 4.0, dated 23 November 2022, decision issued on 14 September 2022). The trial was conducted in full accordance with the Declaration of Helsinki and relevant national data protection regulations (DSGVO).

A prospective clinical trial employing a mixed-methods approach, integrating qualitative and empirical research methodologies was used. This approach enabled a contextualized, process-oriented analysis of patients’ needs over the course of their cancer journey. Grounded theory methodology guided the qualitative component, allowing for inductive theory development based on patients’ lived experiences. The study was conducted between October 2022 and April 2025 at the Institute for History, Theory, and Ethics of Medicine at Johannes Gutenberg University Mainz, Germany, in collaboration with the Departments of Gynecology and Obstetrics and of Internal Medicine at the University Medical Center Mainz.

### Recruitment and participants

Participants were recruited from affiliated oncology departments at the University Medical Center Mainz at the point of therapy initiation, transition into or during maintenance therapy, following the finalization of individualized treatment plans by the respective interdisciplinary tumor boards. Recruitment took place once the therapeutic regimen had been formally confirmed and prior to the baseline research visit. All participants provided written informed consent after receiving detailed information about the study’s objectives, procedures, and their rights as research participants. They were explicitly informed of their right to withdraw from the study at any time without providing a reason and without any consequences for their ongoing medical care. The study was conducted in accordance with the European General Data Protection Regulation (GDPR/DSGVO) and institutional ethical standards.

A total of 20 participants were enrolled, all of whom had received a histologically confirmed diagnosis of a gynecologic malignancy or breast cancer and were either preparing to initiate therapy, to change therapy regimen or actively undergoing maintenance therapy. Each participant was interviewed using a semi-structured interview guide that was developed based on a framework derived from existing literature. The structure was applied flexibly to accommodate the unique clinical and emotional circumstances of each participant resulting in a total of 20 semi-structured interviews conducted between December 2023 and April 2025.

While the sample size (*n* = 20) is modest and includes both gynecologic and breast cancer cases, this reflects the exploratory and qualitative nature of the study. The aim was not statistical representativeness, but rather to capture a heterogeneous range of patient experiences and identify common as well as divergent needs. This deliberate heterogeneity allowed us to trace shared ethical dimensions across different oncologic contexts. Generalizability is therefore limited, and the findings should be understood as hypothesis-generating and as a validation study within the European care setting.

### Eligibility criteria

Participants were eligible for inclusion if they were adult women (> 18 years) with a histologically confirmed initial diagnosis of gynecologic or breast cancers (including ovarian, cervical, uterine, or breast see Table 1). All participants were required to have full decisional capacity and the ability to provide informed consent. Patients currently undergoing treatment for a psychiatric disorder or those with cognitive impairments that might interfere with consent or meaningful participation in the interview process were excluded. Additionally, patients of advanced age (> 75 years) whose treatment was guided primarily by geriatric oncology assessments and age-specific therapeutic goals were not eligible, in order to ensure consistency in the clinical decision-making frameworks examined across the study cohort. Participants were undergoing standard oncologic care that included surgical and/or systemic treatments (e.g., chemotherapy). Because this was an exploratory qualitative study, we did not stratify analyses by modality, and we explicitly acknowledge this as a limitation.

**Table 1:** Eligibility criteria.

Inclusion criteria	Exclusion criteria
Female	Treatment for psychiatric diagnosis
Age > 18	age > 75 years and treated on basis of gerontological oncology assessments/age-appropriate treatment goals
Histologically confirmed gynecologic or breast cancer (ovarian, cervical, uterine or breast)	
Capable to give consent	

### Data generation

Data was collected through semi-structured, in-depth interviews guided by a thematically structured interview framework developed specifically for this study (Supplementary file 1). The guide encompassed three major domains: (1) bodily and physical experiences of illness, (2) individual therapy goals and treatment-related decision-making, and (3) expectations—both positive and negative—regarding social participation, identity, and self-concept. The interview structure was designed to facilitate narrative exploration, with open-ended questions and flexible prompts tailored to the participant’s clinical and emotional context.

All interviews were conducted in person by the lead interviewer, a clinician-researcher and board-certified gynecologist, distinct from the participants’ attending oncologist. Her dual role as both practitioner and researcher fostered a strong rapport with participants and enabled in-depth discussions of clinically sensitive issues such as side effects, sexuality, fertility, and long-term health priorities. This shared background often enhanced participants’ willingness to engage in detailed accounts of their treatment experiences. The interview guide was inspired by established qualitative frameworks and adapted iteratively in line with grounded theory principles, allowing emerging themes to shape subsequent interviews and questioning strategies.

Interviews were conducted in a private, secure and calm setting with the interviewer and participant present. Each interview was a standalone session without follow-up. Duration of the interviews varied between 15 and 52 min with a mean duration of 32 min. Each interview was audio-recorded using a digital recording device, with written informed consent obtained in advance. The recordings were transcribed verbatim and pseudonymized during the transcription process to ensure participant confidentiality. In addition to the recordings, field notes were documented after each interview to capture non-verbal cues, emotional responses, and contextual details that might inform later stages of analysis. The interviewer kept reflexive notes after each session to monitor potential biases.

### Data analysis

The interview transcripts were analyzed using principles of grounded theory methodology, following the iterative and comparative logic outlined by Charmaz et al. 2017^28^. This approach enabled the development of categories and conceptual insights directly rooted in participants’ narratives, with a particular focus on their perceptions of needs, personal treatment goals, and psychosocial impacts of cancer and therapy.

Transcripts were uploaded and coded using MAXQDA Analytics Pro (VERBI Software, 2022), a qualitative data analysis software that facilitated systematic coding, memo writing, and comparative data organization. The analysis proceeded in three stages:

1. Initial (open) coding, where transcripts were read line-by-line and coded inductively to capture participants’ language, metaphors, and expressed meanings by three assigned members of the research team;
2. Focused coding, in which the most significant and frequently occurring initial codes were refined into more conceptual categories;
3. Theoretical coding, where relationships between categories were examined to construct higher-order themes and integrative theoretical insights.

To enhance credibility, three researchers independently coded all transcripts and resolved discrepancies in consensus meetings. Supported by systematic memo-writing, analytic decisions were discussed in regular team sessions. Transferability was supported by thick description of setting and participant characteristics. Dependability was promoted by maintaining an audit trail in MAXQDA (timestamped memos, iterative codebook versions) documenting procedural and analytic changes. Confirmability was addressed through reflexive journaling by the lead interviewer and documentation of coding decisions and consensus rationales to minimize idiosyncratic interpretations. Analysis continued until ten subjects in the initiation phase of their oncological therapy and ten others in the maintenance or escalation phase were included. Theoretical saturation - that is, when additional interviews no longer yielded new conceptual categories or altered the understanding of emerging themes - was reached after 7 interviews in initiation phase and after 8 interviews in maintenance/escalation phase. The grounded theory approach allowed the resulting categories to remain closely aligned with patients’ perspectives while also generating theoretical contributions concerning the practice and perception of needs in gynecologic oncology.

After focused coding, we grouped “needs” codes into inductive domains. We then conducted a structured matrix comparison against the eight Picker-Gerteis patient-experience domains, organizing a matrix (rows = inductive domains; columns = Picker-Gerteis domains) and populating cells with representative codes and exemplar quotations. Proposed mappings were reviewed in team consensus meetings documented through analytic memos. For each inductive domain, we classified the relationship as either an overlap (concordant) with existing domains or an addition (extension) where content was not adequately captured or required temporal/ethical refinement.

## Results

A total of twenty female participants, aged between 31 and 72 years (mean age 49.4 years), were included in the gynecologic cohort of the study. The majority were married (65%), while 10% were divorced and 25% single. 60% of the participants reported having children, with average of 1.3 children per participant (range: 1 to 4). In terms of educational attainment, 20% had a high school diploma, 40% had an intermediate school-leaving certificate, 30% had completed university levels, while 5% had a primary school or technical college qualification.

The qualitative coding process yielded a total of 952 codes through open coding, which were subsequently organized into 11 overarching categories via focused coding. These categories encompassed physical effects, individual restrictions, treatment setting, coping strategies, aims, emotional reactions, social participation, body image, self-image, as well as „quotable passages“ and „others“. Several of these categories were further refined into subcategories, ranging from none (e.g., self-image) to as many as 11 subcategories (e.g., emotional reactions).

For the present analysis 78 codes within the data (besides the previously named overall categories) were identified that directly related to needs and allocated to five subdomains: social (*n* = 21), cognitive (*n* = 21), system-related (*n* = 15), emotional (*n* = 14) and physical (*n* = 7). Codes for domain needs were present in 95% (*n* = 19) of the interview transcripts.

**Fig. 1:**
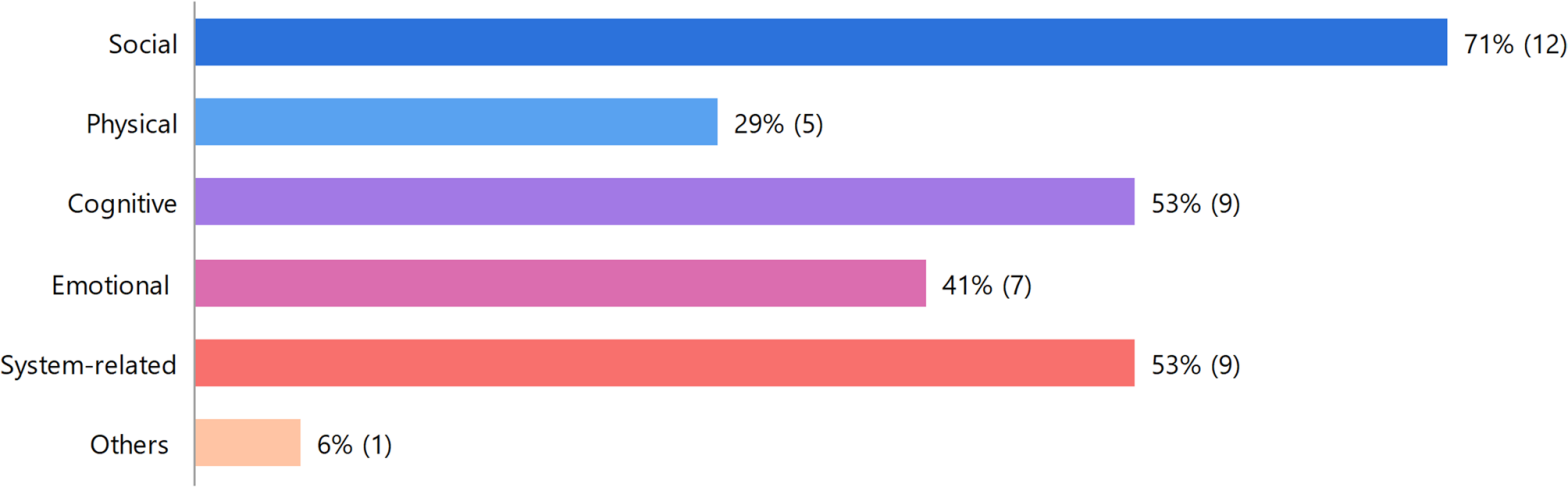
Proportional distribution of the identified subdomains related to needs in presented cohort.

Figure 1 illustrates the proportional distribution of the identified subdomains related to needs.

### Subdomain social needs

Social needs were expressed by 71% of the participants.

Participants in general emphasized the focal role played by family and friends as a source of love and cordiality. Mainly, participants mentioned the need for support in their daily lives from their social environment such as assistance with childcare and looking after children or pets, but also with shopping or company to medical appointments.

Maintaining normality was important, with many preferring social interactions not centered on illness: “*When we see each other*,* we don’t always talk about the illness… I don’t need to talk about it every time*” (Gyn_7). Several emphasized their rejection of pity (Gyn_9). Participants also expressed that being needed (e.g. by kids or pets) was important to them to maintain normality. It was also shown that they favored support of close confidants such as their partner, “my husband” (Gyn_17), over professional support.

At the same time, ambivalences in social interactions became apparent, often characterized by changed social roles (or at least perceived changes in social roles) due to the illness (Gyn_5, Gyn_12). One participant mentioned that, after chemotherapy, although being bodily present, her husband and children “already know that Mum is useless for now” (Gyn_12).

### Subdomain cognitive needs

Cognitive needs were expressed by 53% of the participants. Central themes included the need for confidence and reliable information. One participant described “*the light at the horizon… this straw*,* no matter how small it is*” (Gyn_1), while others emphasized that being fully informed reduced anxiety, whereas uncertainty heightened distress (Gyn_11, Gyn_13).

Recognition by doctors and being valued as individuals were highlighted (Gyn_16). Control and security were also important: some participants wanted to pre-plan aspects of their care or preferred more aggressive interventions (Gyn_17). However, most accepted a guided approach, trusting their doctors’ expertise (Gyn_7, Gyn_11). A minority preferred limited information, underscoring diverse needs for involvement and knowledge: *“I think it was actually a good thing that I didn’t know in advance exactly what was going to happen”* (Gyn_17). Others even explicitly verbalized that they had a right not to know and chose to exercise it.

Other cognitive needs for control and security were also expressed. One participant stated that she needed control to feel at ease. This even extended to pre-planning her own funeral and obituary. However, she emphasized that this was not about giving up (Gyn_17).

### Subdomain emotional needs

Emotional needs were expressed by 41% of participants, often overlapping with social aspects. While some desired professional psychological support, others found minimal sessions sufficient (Gyn_10, Gyn_19). Emotional support from family members, especially children, was frequently emphasized (Gyn_17).

Participants also described the importance of time alone to process their diagnosis (Gyn_6). Empathetic communication from medical staff was valued, particularly for those with anxiety (Gyn_12). Warmth and humanity were repeatedly described as essential: “*This person is not only well-informed but also genuinely warm-hearted… Every time I come here… I know the people are very*,* very kind*” (Gyn_6).

Finally, participants emphasized the need to preserve independence and autonomy, valuing encouragement to remain active agents in their own illness narratives (Gyn_14, Gyn_16).

### Subdomain system – related needs

System-related needs were reported by 53% of participants. Common concerns included long waiting times, difficulties reaching specialized staff, and fear of treatment delays (Gyn_5, Gyn_9). Experiences of errors or uncertainty about treatment necessity sometimes created mistrust in the healthcare system (Gyn_8, Gyn_18).

Participants frequently expressed the need for reassurance and continuity of care, especially during chemotherapy or when questioning whether to continue therapy (Gyn_8, Gyn_20). Trust and safety were recurring themes, as were the desire to feel seen and acknowledged by healthcare professionals: “*I would have wished for some reassurance - that what I was experiencing was painful*,* but not unusual. And to be seen*” (Gyn_5). Several voiced the wish for a more holistic care approach and a consistent point of contact to avoid fragmented communication (Gyn_18).

### Subdomain physical needs

Physical needs were expressed by 29% of participants. Key themes included the need for rest, moderate physical activity, and relief from social responsibilities. Some participants initially struggled to accept bodily changes such as scars (Gyn_17), while others preferred radical surgical interventions to remove affected organs.

Regaining trust in their own bodies was central to future planning. For younger participants, this was linked to concerns about fertility and family planning: “*At present*,* I cannot envision having children… We first require additional years of MRI monitoring*” (Gyn_16).

## Discussion

Our findings resonate with and extend the eight domains of patient-centered care outlined by Gerteis and colleagues in *Through the Patient’s Eyes*^5^. These domains - respect for personal values, clear information and education, emotional support, involvement of family and friends, physical comfort, coordinated care, continuity, and access to treatment - have served as a patient-informed benchmark for quality care. Rather than applying this framework directly, our qualitative analysis uses it as a reflective lens to assess whether, and how, patients’ reported needs align with these domains, and to identify areas where the current model may require further ethical or temporal refinement.

*Needs Vary by Phase: The Importance of Timing*.

One of the clearest contributions of our study is the insight that patient needs are phase-dependent, with the initiation phase - the period immediately following diagnosis and treatment planning - emerging as the most intense and complex. Patients in this stage reported cognitive disorientation, emotional overload and a need for structured orientation. As one participant shared, *“Right at the beginning [.] I’m the kind of person who*,* if I know everything*,* I stay calm. But the moment there’s uncertainty*,* I can’t handle it”* (Gyn_11), underlining the urgency of coherent information delivery. Another noted, *“I just needed someone to say: It’s serious*,* but it’s not extraordinary. Just help me make sense of it”* (Gyn_5). While these concerns fit within the domains of information and emotional support, our data suggest that these needs are not static - they peak early and diminish over time. This temporal aspect is largely underrepresented in the original Picker-Gerteis framework, yet it aligns with recent international calls for more phase-sensitive approaches to PCC in oncology^22^.

*Emotional and Social Needs: Recognition beyond Empathy*.

Our findings strongly support the domain of emotional support, yet they add moral depth by revealing how emotions are tied to issues of dignity, identity, and stigma. Patients frequently resisted pity, minimized disclosure, and struggled with the tension between vulnerability and self-presentation. As one stated, “*I didn’t want anyone’s pity. That was really important to me*” (Gyn_9), illustrating a desire for respect grounded in social recognition rather than sympathy. This complements existing work on cancer stigma^23–25^, but also complicates the Picker-Gerteis domain by suggesting that emotional needs must be understood relationally and ethically, not just psychologically.

Following Goffman’s analysis of stigma and recent oncology policy debates, resistance to pity and efforts to preserve normality function as claims to recognition and dignity, not just as “support needs”^24,25^. Similarly, social support was valued not only in terms of companionship or caregiving, but as a way to preserve roles, autonomy, and mutual recognition. This affirms the relevance of family and relational involvement, but also points to the need for a more nuanced understanding of relational autonomy - particularly for women navigating care responsibilities, body image, and changing roles.

*Cognitive Needs: Epistemic Balance and Moral Agency*.

The domain of information and education is well supported by our data but requires expansion. Several participants described needing not just knowledge, but meaningful framing, validation, or even the right not to know. These cognitive needs are ethically complex. Patients positioned themselves as moral agents seeking epistemic balance - between clarity and overwhelm, between guidance and independence. This aligns with theories of epistemic justice^26^ and highlights that how information is offered is as ethically significant as what is shared. Internationally, studies show that trust in physicians and treatment decisions depends less on the quantity of information and more on the quality of relational framing^17,18^.

*System-Related Needs: From Logistics to Moral Trust*.

Patients frequently described system-level frustrations - such as fragmented care, lack of continuity, or unclear responsibilities - not just as logistical inefficiencies, but as violations of trust and safety. One participant explained, “*There was no one with an overview - just the chemo part here*,* but no one to talk to about the surgery or aftercare*” (Gyn_18). While these concerns map onto the Picker-Gerteis domains of coordination, continuity, and access, our data suggests they carry an additional moral weight. Failures in continuity were experienced as abandonment, poor coordination as disempowerment. These findings suggest a need to think about structural compassion and care systems designed not only for efficiency but for psychological and moral containment. This resonates with recent debates on the limitations of guideline-driven oncology, where strict adherence to standardized protocols can conflict with the ethical imperative to preserve trust and individualized care^18,19^. Read through the NAM/IOM quality lens, these are not only coordination/continuity issues but matters of moral trust and structural compassion^3,18,19^.

*Physical Needs: Embodied Meaning and Future Planning*.

Though less frequently emphasized, physical comfort was linked to deeper experiences of bodily integrity, identity, and self-image. For women facing fertility loss, surgical scars, or visible changes, physical needs were inseparable from moral concerns about femininity, future planning, and selfhood. This lends further ethical dimension to the Picker-Gerteis framework, reinforcing that physical care is not morally neutral - it is part of restoring dignity and enabling agency.

Overall, by juxtaposing patients’ narratives with the Picker-Gerteis domains, our findings affirm many of the framework’s core values - while also identifying gaps and ethical intensities that are underarticulated. Patient-centered care is not merely about aligning services with preferences; it is about responding to moral and relational vulnerabilities that shift over time. Taken together, these observations justify an explicitly ethical reading of needs alongside the experiential domains of patient-centered care. Our findings contribute three key additions:

1. Temporal sensitivity: Needs are not stable - they intensify at critical moments (e.g., diagnosis) and taper over time.
2. Ethical depth: Patients’ needs are not just practical or emotional - they are grounded in social identity, epistemic agency, and moral distress.
3. Interdependence of domains: Emotional, cognitive, and systemic needs are rarely experienced in isolation, suggesting that PCC must address the intersections of these domains, not just their individual presence.

In summary, the Picker-Gerteis framework provides a valuable reference point for understanding what patients prioritize in their care experiences. Our findings largely support its eight domains, while also suggesting areas where additional nuance may be helpful. Specifically, the data highlight how patient needs are shaped by the phase of illness, with particular intensity during the initiation phase. Furthermore, many needs - especially in the emotional, cognitive, and systemic domains - were experienced as interdependent and ethically significant. These insights suggest that effective PCC requires attention not only to the content of care but also to its timing, context, and moral dimension. By grounding these domains in the lived experiences of women undergoing cancer treatment, our study offers a context-specific contribution that complements and extends existing models of patient-centered care.

Summarized, our findings contribute to an emerging international conversation on patient-centered care in oncology. While recent studies from North America, Australia, and Asia have advanced the conceptual and practical dimensions of PCC^16,22,27^, ethically nuanced, European-based qualitative data on women’s needs in gynecologic oncology remain scarce. By situating our analysis within a German and broader European healthcare context, this study provides a preliminary validation of ethically salient patient needs that complements existing international evidence and addresses this important gap.

*Strengths and limitations*.

While several studies on PCC and patient needs have been conducted in the United States and Canada, there remains a paucity of research in the European context. Moreover, existing literature does not sufficiently address the specific needs of gynecologic oncology patients. Focusing on a specific tumor subgroup allows for a detailed, needs-oriented analysis, however, it simultaneously limits the transferability and generalizability of the findings to other cancer populations.

Additionally, the study was conducted at a single university medical center, which may have introduced context-specific factors that constrain the applicability of the results beyond this setting. Due to the selected tumor types, a potential gender bias cannot be ruled out. Furthermore, participant bias may have influenced the findings, as patients seeking treatment at a university hospital might be more open to research participation and experimental therapies. Similarly, interpretative bias - an inherent challenge in qualitative research - cannot be completely excluded.

A key limitation of the study lies in the relatively small and heterogeneous cohort (*n* = 20), which included both gynecologic and breast cancer patients without stratification by tumor type, prognosis, or treatment modality. This limits generalizability. The findings should therefore be understood as exploratory and as providing preliminary validation of ethically relevant needs, which require further investigation in larger, stratified samples.

Despite these limitations, the study offers valuable preliminary insights into the ethically salient needs of women with gynecologic cancers within a European healthcare context. These findings underscore the necessity of embedding ethically responsive, context-sensitive practices within routine oncological care - an area that remains underrepresented in international PCC research^22,23^.

*Implications for practice*.

Our findings indicate that women’s needs are phase-specific, ethically charged, and interdependent rather than siloed. We treat “timing” (phase sensitivity) and “ethical depth” (epistemic agency, recognition, moral trust) as explicit design principles for care processes, training, and organizational policy. Translating these insights into routine oncologic care requires changes at the level of clinical encounters, team competencies, and service design.

1. Protect time for communication at critical moments

Time pressure is a structural barrier to patient-centered care. We recommend designating protected clinical time for communication and orientation, especially at treatment initiation (diagnosis and first plan discussion) and at predictable milestones (e.g., treatment change, escalation/de-escalation, and transition to survivorship or palliative focus). These encounters should have explicit goals (orientation, framing of options/uncertainties, eliciting values and preferences, safety-netting) and be documented in the care plan. The time devoted to communication is as integral to the patient experience as the time devoted to therapy.

2. Provide phase-sensitive, ethically aware information

Information needs peak early and taper later. Teams should (a) front-load coherent, jargon-light information at initiation; (b) revisit key decisions briefly at milestones; and (c) respect epistemic agency—including patients’ right not to know or to pace information. Short, structured summaries (“what we know/what it means/what to watch for/next step”) and explicit checks for understanding help reduce anxiety and moral distress.

3. Ensure continuity and coordinated contact

Patients frequently experienced fragmentation as a threat to trust and safety. Services should assign a consistent point of contact (e.g., nurse navigator or care coordinator) who tracks the plan across outpatient, inpatient, and supportive services and who can proactively close loops after tumor-board decisions, imaging, or therapy changes.

4. Attend to relational and social needs, not only symptoms

Support that preserves roles, dignity, and independence (e.g., practical assistance, partner/family inclusion as desired, stigma-aware communication that avoids pity) should be integrated alongside symptom control. Brief screening for social load (caregiving, childcare, work) can trigger targeted support or referrals.

5. Build communication and ethics competencies across training pathways

To sustain these practices, we recommend structured communication training and ethics-sensitive PCC content in undergraduate, postgraduate, and continuing education for oncology teams (physicians, nurses, allied professionals). While our data are from Germany, training provision varies across Europe, underscoring the need for curricular integration (e.g., shared decision-making, dignity-affirming language, handling uncertainty, discussing fertility/body image, and managing the right not to know). Team-based simulation, observed practice with feedback, and reflective debriefing can reinforce skills.

6. Embed structural, guideline, and policy supports

Service-level policies should (a) allocate scheduled communication appointments at initiation and milestones; (b) standardize brief follow-up touchpoints (in-person or telehealth); (c) document communication preferences (content, pace, involvement of family) in the electronic health record (EHR); (d) include PCC indicators (continuity, timely callbacks, clarity of plan) in quality dashboards; and (e) incorporate these steps into local pathway/guideline checklists and commissioning/contracting requirements to protect time and continuity.

Next steps for evaluation: Because our qualitative, heterogeneous cohort was not stratified by tumor type or prognosis, larger, stratified studies are warranted to test how phase-specific and disease-specific needs vary and to evaluate whether the above measures improve trust, orientation, and patient-reported outcomes. Future work should include multicenter European cohorts with stratification (tumor type, biology/metastatic status, prognosis), cross-cultural comparisons, longitudinal designs to map temporal change, and interventional studies testing whether protected time, a designated contact, and phase-sensitive information improve trust, orientation, and patient-reported outcomes.

## Conclusion

Existing models of patient-centered care identify core domains—respect for personal values, clear communication and education, emotional support, involvement of family and friends, physical comfort, coordinated care, continuity, and access. Our qualitative analysis of women receiving treatment for gynecologic or breast cancer refines this framework by adding an explicitly ethical and phase-sensitive perspective grounded in patients’ lived experience.

Notably, needs vary by illness phase and shift over time. We interpret these patterns as ethical claims: to epistemic agency at initiation (calibrated information, including the right not to know), to recognition and dignity during adaptation (avoiding pity, preserving roles), and to moral trust through continuity and coordination. Practically, this argues for a temporal-and-ethical implementation of PCC: a protected initiation consultation with front-loaded, plain-language orientation; brief milestone micro-consults; a designated point of contact to ensure continuity; and routine documentation of communication preferences in the care plan, supported by ethics-sensitive communication training.

While our single-center, small, heterogeneous cohort limits generalizability, it provides a preliminary validation of ethically salient, phase-dependent needs within a European setting and delineates testable process changes. Future work should include larger, stratified, multicenter and longitudinal studies, alongside pragmatic evaluations assessing effects on orientation, trust, decisional confidence, and continuity.

## Supplementary Information

Below is the link to the electronic supplementary material.

Supplementary Material 1

## Data Availability

The datasets generated and analysed during the current study are not publicly available due to data protection and until final evaluations have been completed. They are available from the corresponding author in pseudonymized form on reasonable request.

## Abbreviations

GDPR/DSGVO: European General Data Protection Regulation
Gyn_X: Number of interview performed in gynecology
PCC: Patient centred care
NAM: National Academy of Medicine
IOM: Institute of Medicine
DRKS: Deutsche Register Klinischer Studien (German Clinical Trials Register)
